# Record Index–Bandgap Trade‐Off: CdPS_3_ as a High‐Index van der Waals Platform for Ultraviolet–Visible Nanophotonics

**DOI:** 10.1002/nap2.70142

**Published:** 2026-06-03

**Authors:** Maksim R. Povolotskiy, Aleksandr S. Slavich, Georgy A. Ermolaev, Dmitriy V. Grudinin, Nikolay V. Pak, Ilya A. Zavidovskiy, Konstantin V. Kravtsov, Anton A. Minnekhanov, Mikhail K. Tatmyshevskiy, Alexander V. Syuy, Dmitry I. Yakubovsky, Arslan Mazitov, Liudmila A. Klimova, Adilet N. Toksumakov, Alexander V. Melentev, Elena Zhukova, Davit A. Ghazaryan, Gleb Tselikov, Ivan Kruglov, Sergey M. Novikov, Andrey A. Vyshnevyy, Aleksey V. Arsenin, Kostya S. Novoselov, Valentyn S. Volkov

**Affiliations:** ^1^ Moscow Center for Advanced Studies Moscow Russia; ^2^ Emerging Technologies Research Center, XPANCEO Dubai UAE; ^3^ Laboratory of Advanced Functional Materials Yerevan State University Yerevan Armenia; ^4^ National Graphene Institute (NGI), University of Manchester Manchester UK; ^5^ Department of Materials Science and Engineering National University of Singapore Singapore Singapore; ^6^ Institute for Functional Intelligent Materials, National University of Singapore Singapore Singapore

**Keywords:** cadmium phosphorus trisulfide, high refractive index, integrated photonics, optical constants, waveguides

## Abstract

The development of nanophotonics is hindered by a fundamental trade‐off between a material's refractive index (*n*) and its electronic bandgap (*E*_*g*_), which severely restricts the choice of materials for short‐wavelength applications. This challenge is particularly acute in the visible and ultraviolet (UV) spectra, where high‐performance devices require materials that are simultaneously highly refractive and transparent. Here, we report on the van der Waals (vdW) crystal cadmium phosphorus trisulfide (CdPS_3_) as a solution to this long‐standing problem. Through comprehensive optical and structural characterization, we show that CdPS_3_ possesses an anomalously high in‐plane refractive index across the visible spectrum approaching three in the near‐UV, combined with a wide indirect bandgap. This combination of properties is validated by first‐principles calculations and direct near‐field imaging of highly confined waveguide modes. These findings establish CdPS_3_ as a leading material for UV–visible photonics, opening a new pathway for the development of high‐density integrated circuits and metasurfaces.

## Graphical abstract

The fundamental index–bandgap trade‐off strictly limits materials for short‐wavelength nanophotonics. The van der Waals crystal CdPS_3_ emerges as a compelling alternative platform challenging this paradigm. Combining an exceptionally high refractive index approaching 3 with a broad mid‐infrared‐to‐ultraviolet transparency window, CdPS_3_ unlocks new potential for ultra‐dense photonic integrated circuits.

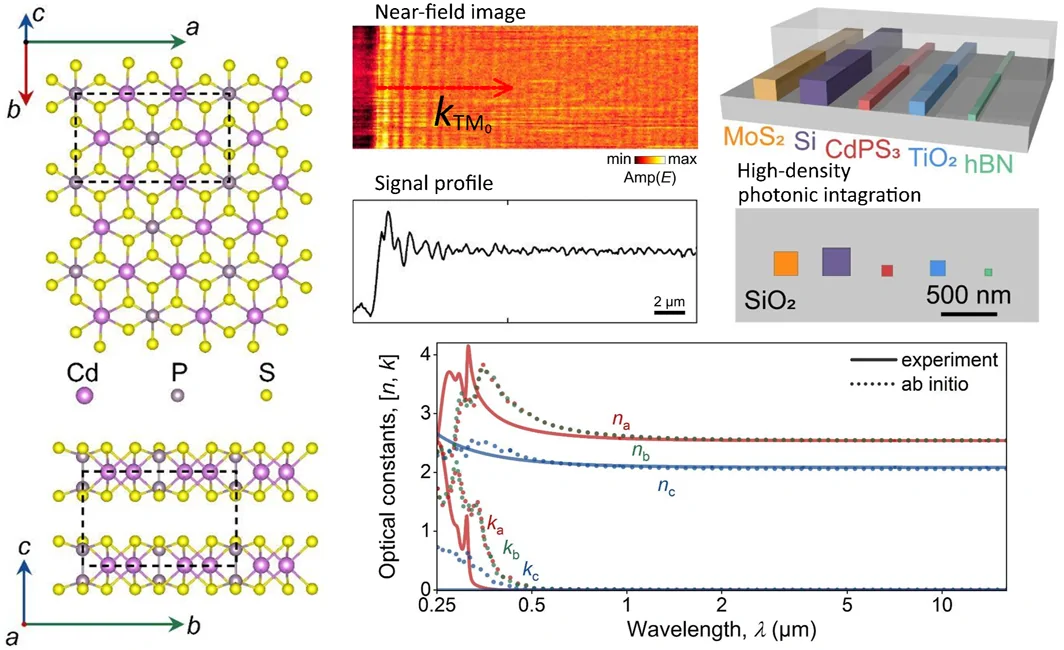

## 1 Introduction

The ongoing paradigm shift in nanophotonics from lossy plasmonics to all‐dielectric platforms has intensified the search for materials that combine a high refractive index with low optical loss, particularly in the visible and ultraviolet (UV) spectral ranges [1, 2, 3]. Progress toward this goal has been restricted by a materials bottleneck: The lack of materials that offer both a very high refractive index for strong light confinement and a wide bandgap for low‐loss operation at short wavelengths. This trade‐off, described by Moss's law [4], has forced compromises. Silicon offers a high index but is opaque in the visible [5], whereas wide‐bandgap materials such as TiO_2_ and hBN are transparent but have relatively modest refractive indices [6].

Van der Waals (vdW) materials have been explored as a solution [7, 8, 9, 10, 11, 12], with TMDCs showing high indices in the near‐ and mid‐infrared or hBN and GeS_2_ showing UV transparency [6, 13]. However, a critical gap exists for a vdW material combining both properties for visible/UV photonics [14]. The metal phosphorus trichalcogenide (MPX_3_) family has recently emerged as a promising new platform [15, 16, 17, 18, 19, 20, 21, 22], with CdPS_3_, in particular, having been demonstrated in solar‐blind UV photodetectors, hinting at its suitable wide bandgap of 3.1–3.5 eV [23, 24, 25].

However, to truly assess if CdPS_3_ can solve the material challenge for short‐wavelength nanophotonics, a complete and accurate measurement of its broadband optical constants is essential, but it has not been reported yet. Without knowing the full dielectric tensor, it is impossible to accurately model critical performance metrics such as waveguide mode confinement, integration density limited by crosstalk [26], or the efficiency of metasurface elements. This leaves a critical gap between the material's initial promise and its practical implementation in the very technologies it could transform, including advanced integrated systems [6, 8].

In this paper, we directly addressed this application‐critical need. We report a comprehensive measurement of the complete broadband (250–16000 nm) dielectric function of the vdW crystal CdPS_3_. We reveal that it possesses an exceptionally high refractive index coexisting with a wide transparency window, addressing the materials challenge for short‐wavelength photonics. We validate these optical constants through first‐principles calculations and near‐field imaging of highly confined waveguide modes. We used these optical constants to model the performance of CdPS_3_‐based waveguides, showing their potential for achieving unprecedented integration densities. Our work provided the missing foundational data required to unlock CdPS_3_ as an enabling material for a new generation of high‐performance short‐wavelength photonic devices.

## 2 Results

### 2.1 Crystal Structure and Vibrational Response of van der Waals CdPS_3_

Cadmium phosphorus trisulfide (CdPS_3_) is a layered crystal with a monoclinic lattice belonging to the C2/m space group. Its lattice parameters are *a* = 6.218 Å, *b* = 10.763 Å, *c* = 6.867 Å, and *β* = 107.58° [27]. The unit cell consists of two layers, held together by weak van der Waals (vdW) forces. As demonstrated in Figure 1a, each cadmium (Cd) atom within a layer is coordinated by six sulfur (S) atoms in an octahedral arrangement, whereas phosphorus (P) atoms form P–P pairs at the center of P_2_S_6_^4−^ cluster anions. The resulting pattern represents a honeycomb‐like lattice of Cd atoms and P_2_S_6_ groups. The lower picture in Figure 1a represents the layered structure of the crystal.

**FIGURE 1:**
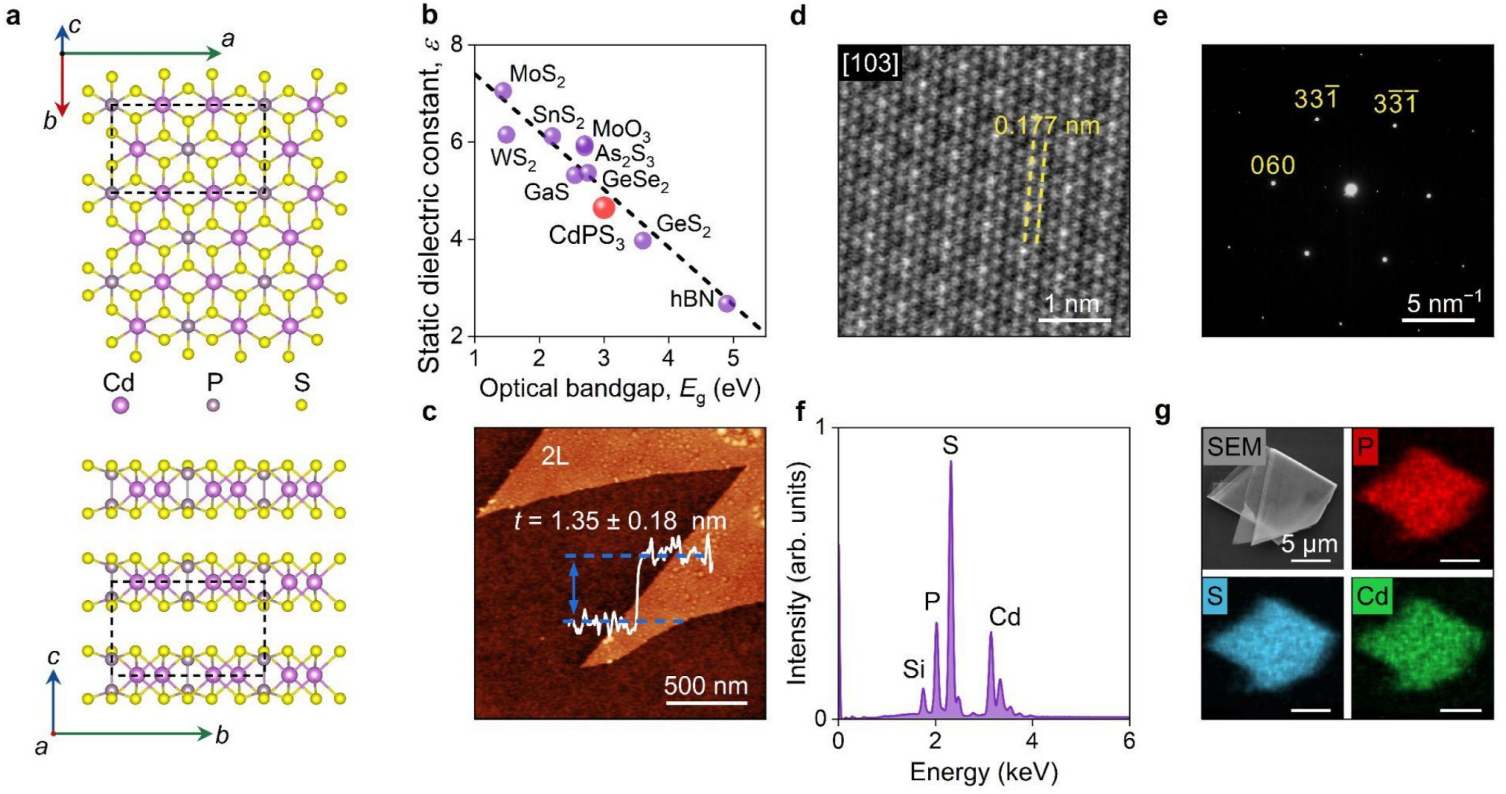
Crystal structure and characterization of CdPS_3_. (a) The lattice structure of CdPS_3_: The top view of an individual monolayer along the [103] direction of the bulk structure and the side view along *a*‐axis. (b) Calculated out‐of‐plane static dielectric constants of van der Waals materials plotted against the experimental optical bandgaps that is, the light energy where a material starts absorbing light [13]. For calculation details see Methods. (c) Atomic force microscopy image of an exfoliated bilayer CdPS_3_ flake. (d) A high‐resolution HAADF‐STEM image of CdPS_3_ flake. (e) SAED pattern characteristic of CdPS_3_ showing the main reflections associated with reciprocal lattice vectors. (f) Energy‐dispersive X‐ray spectrum (EDX) of the CdPS_3_ flake transferred onto Si substrate. SEM image of the flake and EDX elemental mapping of corresponding elements: P in red, S in blue and Cd in green.

As illustrated in Figure 1b, a plot of calculated static dielectric constant versus experimental optical bandgap shows that CdPS_3_ fills a critical gap in the available material landscape. It is positioned between TMDCs, which are primarily functional in the IR, and wide‐bandgap materials such as hBN and GeS_2_, which stand out in the UV. The weak vdW forces facilitate easy mechanical exfoliation into thin flakes, down to a few monolayers (see Figure 1c). The number of layers was estimated through comparison of the measured height (1.3 nm) and theoretical unit cell dimensions (0.63 nm in out‐of‐plane direction). Nevertheless, in this work we focus on properties of bulk (thickness > 50 nm) flakes and their potential applications in nanophotonics.

To verify the crystal quality of the CdPS_3_ flakes, we exfoliated them onto a porous SiN_x_ membrane and characterized their structure using scanning transmission electron microscopy (STEM). Figure 1d shows a high‐angle annular dark‐field (HAADF) image of the transferred flake. The observed lattice spacings would correspond to the known crystallographic planes, confirming that the exfoliated flakes retain the bulk crystal structure. The homogeneous quality of the CdPS_3_ flakes is evidenced by the sharp regular pattern that would be seen in a selected area electron diffraction (SAED) image from the same flake. We also validated the chemical composition of the CdPS_3_ flakes via scanning electron microscopy (SEM) combined with energy‐dispersive X‐ray spectroscopy (EDX), as shown in Figure 1f. Figure 1g represents SEM image of a bulk CdPS_3_ flake transferred onto a Si substrate and the corresponding EDX maps of Cd, P, and S elements which confirm the uniform distribution of these atoms throughout the flake. The Cd:P:S (20.2%:20.2%:59.6%) atomic ratio match well with the 1:1:3 stoichiometry of CdPS_3_.

To investigate the vibrational properties and confirm the crystalline symmetry of the CdPS_3_ flakes, we performed Raman spectroscopy measurements. The representative Raman spectrum, acquired with a 632.8 nm excitation laser, is presented in Figure 2a, whereas angle‐resolved polarized Raman intensity map is shown in Figure 2b. The spectrum reveals several distinct phonon modes at Raman shifts of 78, 125, 223, 247, 271, 377, and 564 cm^−1^. The positions and relative intensities of these peaks are in excellent agreement with previously reported spectra for monoclinic CdPS_3_ [28, 29, 30], confirming the high quality and purity of the crystal.

**FIGURE 2:**
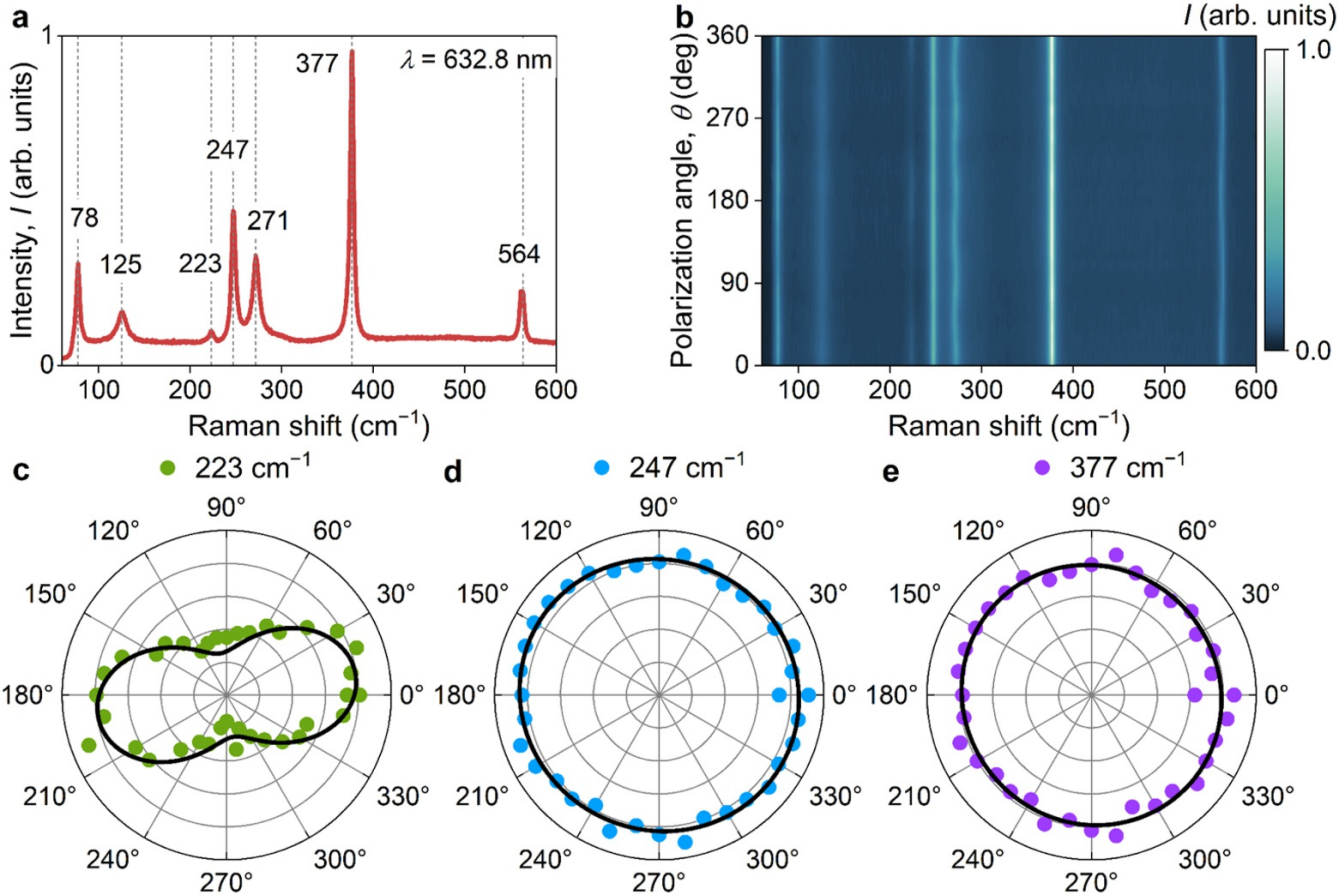
Polarized Raman spectra of bulk CdPS_3_. (a) Raman spectrum of bulk CdPS_3_ at ambient conditions. Dashed lines labeled with numbers denote vibrational mode positions with corresponding wavenumbers in cm^−1^. (b) Angle‐resolved polarized Raman intensity map of bulk CdPS_3_. (c–e), polar plots (color dots) and fittings (dark solid line) of three angle‐resolved normalized Raman intensities.

Polar plots of the Raman peak intensities for three active modes are shown in Figure 2c–e. The fitting procedure of angular dependences is discussed in Supporting Information S1: Note 1. The results reveal both isotropic and anisotropic responses: 247 and 377 cm^−1^ modes are polarization‐independent within experimental uncertainty, whereas 223 cm^−1^ mode exhibits a pronounced anisotropy, consistent with the monoclinic crystal symmetry.

Raman spectroscopy of bulk CdPS_3_ flakes reveals an excitation power‐independent vibrational signature. Furthermore, the flakes exhibit ambient stability over multiple weeks. These characteristics presented in Supporting Information S1: Note 1 highlight the material's suitability for reliable and reproducible analysis, as well as its promise for various applications.

### 2.2 Broadband Optical Properties of CdPS_3_

Despite the structural anisotropy of the monoclinic lattice and pronounced anisotropy of its vibrational modes, cadmium phosphosulfide (CdPS_3_) exhibits nearly isotropic in‐plane optical response. Figure 3a, shows a representative polarization‐resolved transmittance map of a CdPS_3_ flake exfoliated on a Schott glass substrate. Across the visible spectrum (450–850 nm), the transmittance remains virtually independent of the incident light polarization angle (standard deviation of *T* ∼0.3%).

**FIGURE 3:**
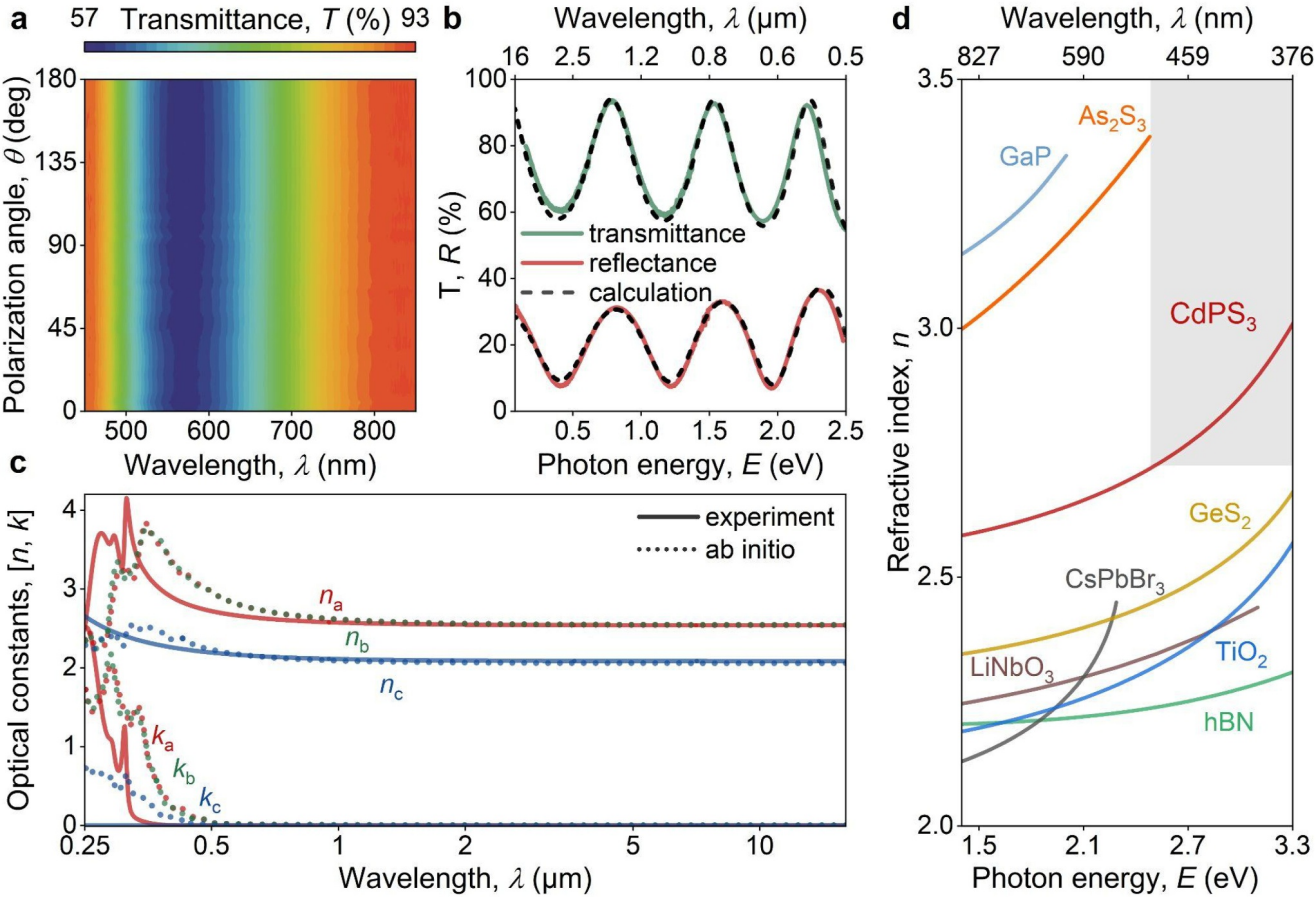
Optical characterization and comparison of CdPS_3_ with other high‐refractive index materials. (a) Polarization‐resolved transmittance map of bulk CdPS_3_ as a function of wavelength and polarization angle. The color gradient illustrates the transmittance percentage across the spectral range (450–850 nm) (b) Experimental reflectance *R* and transmittance *T* (solid curve) compared to calculated spectra (dashed black curve). (c) Refractive index *n* and extinction coefficient *k* of CdPS_3_ crystal measured experimentally (solid curves) and calculated using ab initio methods (dotted curves). The red and green curves correspond to the experimental and calculated in‐plane components, respectively, whereas the blue curves represent the out‐of‐plane components. Experimental data were obtained through spectroscopic ellipsometry measurements. (d) In‐plane refractive index of CdPS_3_ (red curve) compared with high‐refractive index materials within transparency window (*k* < 0.01), including TiO_2_, GaP [31], hBN [6], LiNbO_3_ [32], CsPbBr_3_ [33], GeS_2_ [13], and As_2_S_3_ [34].

To retrieve the dielectric constants over an ultrabroad spectral range (250–16000 nm) with high accuracy, we performed a global analysis combining spectroscopic ellipsometry and normal‐incidence transmittance and reflectance spectra acquired from multiple flakes of different thicknesses. The full spectroscopic analysis procedure is described in detail in the Supporting Information S1: Note 2. As shown in Figure 3b, the representative experimental reflectance *R* and transmittance *T* spectra exhibit excellent agreement with calculations based on the extracted optical constants. Figure 3c presents the derived refractive index (*n*) and extinction coefficient (*k*) for both the in‐plane (*ab*‐plane) and out‐of‐plane (*c*‐axis) directions. The experimental results are strongly supported by ab initio calculations, which replicate the dispersion trends. We used ab initio calculations to support the interpretation of the measured dielectric response, in particular, to confirm its indirect‐gap nature and nearly isotropic in‐plane optical behavior (see Methods and Supporting Information S1: Note 3). The solid curves show the experimentally extracted optical constants, whereas the dotted curves represent single‐shot GW calculations (see Figure 3c). The ab initio results reveal that the in‐plane optical birefringence (*Δn*_ab_) is less than 0.003 in the transparency window (*k* < 0.01). The overall dispersion trends are reproduced well, whereas the remaining deviations, especially in the UV region, are attributed primarily to the absence of excitonic effects in the present GW treatment.

The in‐plane refractive index exceeds *n* = 3 in the visible–near‐UV range and decreases gradually toward the IR, whereas *k*_ab_ remains near zero. Figure 3d contextualizes these findings by comparing the refractive index of CdPS_3_ with established high‐index materials. CdPS_3_ surpasses conventional wide‐bandgap dielectrics such as TiO_2_, LiNbO_3_, and hBN across the visible and near‐UV regions. Furthermore, it offers a significantly wider transparency window than GaP or As_2_S_3_. This unique combination of a high refractive index (*n* > 3) and wide bandgap positions CdPS_3_ as an exceptional material for short‐wavelength nanophotonics, expanding the optical design space traditionally restricted by Moss's rule.

To validate the experimentally determined optical constants (*n*, *k*) and directly confirm the potential of CdPS_3_ for strong light confinement, we used scattering‐type scanning near‐field optical microscopy (s‐SNOM). This technique allows for the real‐space visualization of guided electromagnetic modes within the CdPS_3_ flakes.

We investigated flakes of varying thicknesses exfoliated onto CaF_2_ substrates. When illuminated, light couples into the waveguide modes of the CdPS_3_ flake. The interference between the background signal and the propagating mode results in distinct fringes (Figure 4a). The periodicity of these fringes corresponds to the waveguide mode momentum. Figure 4b shows line profiles extracted from the near‐field maps at *λ* = 700 and 1600 nm for a 254‐nm‐thick‐flake. The corresponding Fourier transforms (Figure 4c) reveal sharp peaks corresponding to the effective indices of the transverse electric (TE_0_) and transverse magnetic (TM_0_) modes. The effective mode indices *n*_eff_ are extracted from these momenta after applying a geometric correction [35, 36], accounting for the oblique incidence angle of the excitation (see Methods and Supporting Information S1: Note 3).

**FIGURE 4:**
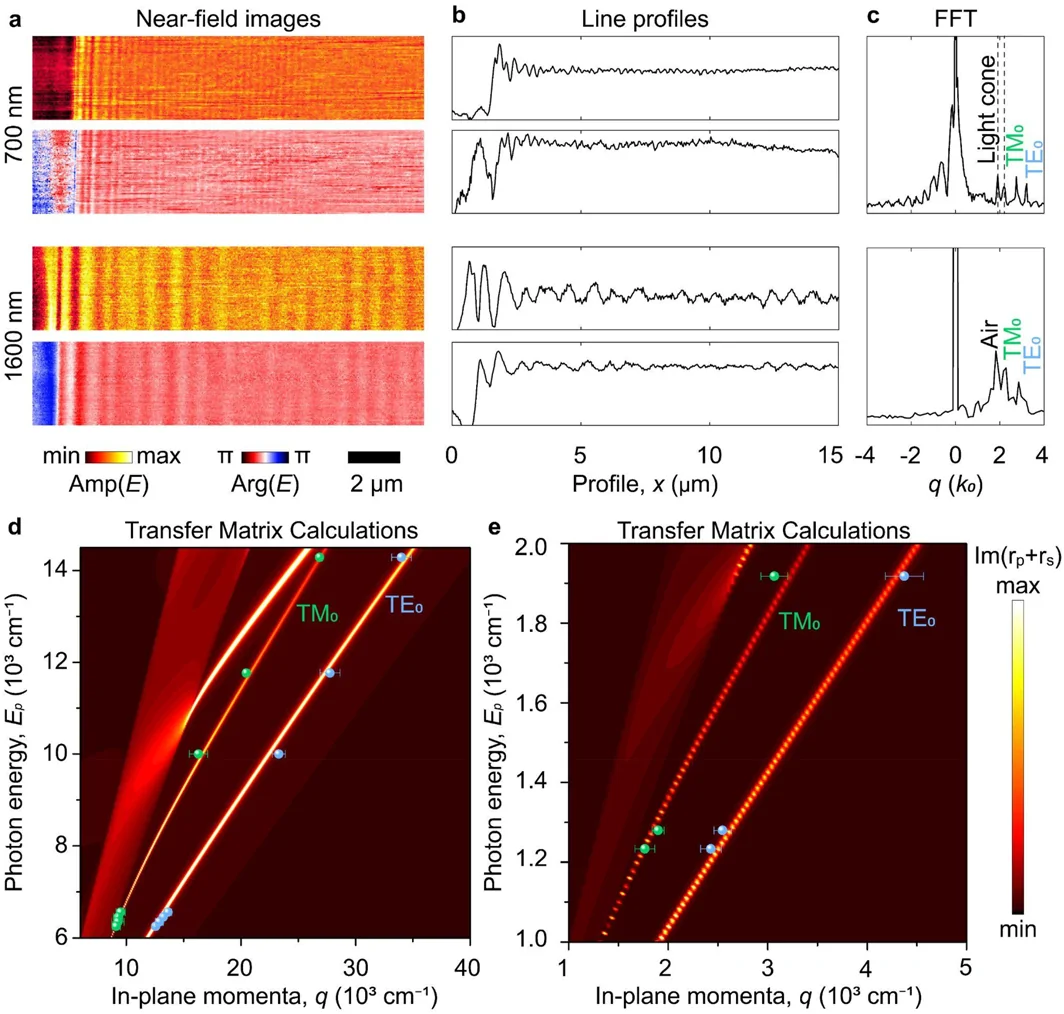
Near‐field analysis of CdPS_3_. (a) Near‐field amplitude amp(E) and phase arg(E) maps of a 254 nm flake taken at 700 and 1600 nm (b) *x*‐line scans taken from panel a and averaged over 3 μm along the *y*‐axis. (c) Complex fast Fourier transform (FFT) of the complex near‐field signal in (b). (d) and (e) Transfer matrix calculations for the Air/CdPS_3_/CaF_2_ system for thickness of the CdPS_3_ 254 and 1200 nm respectively. Green and blue dots show data extracted from the near‐field experiments for the TM_0_ and TE_0_ modes respectively.

We compared these experimental results with theoretical energy‐momentum dispersion relations calculated using the transfer matrix method (TMM) [37], utilizing the optical constants derived from ellipsometry (Figure 3c). The experimental data points for both thin (254 nm, Figure 4d) and thick (1200 nm, Figure 4e) flakes show excellent agreement with the calculated dispersion curves across a broad spectral range (visible to mid‐infrared). This agreement rigorously validates the accuracy of the determined dielectric tensor and confirms the strong optical confinement enabled by the high refractive index of CdPS_3_.

### 2.3 Prospects for High‐Density Photonic Integration

The combination of a high refractive index and low loss at short wavelengths makes CdPS_3_ a compelling platform for high‐density photonic integrated circuits. To quantify this potential, we modeled the performance of CdPS_3_ waveguides and compared them with conventional photonic materials: MoS_2_, GaN, SiN_x_, Al_2_O_3_, Si, TiO_2_, and hBN (see Methods and Supporting Information S1: Note 5). We evaluated the performance at the shortest wavelength (*λ*_min_) where each material maintains high transparency, defined here by an extinction coefficient threshold of *k* = 0.01. This corresponds to 375 nm for CdPS_3_, 370 nm for TiO_2_, 364 nm for GaN, 265 nm for SiN_x_, 230 nm for Al_2_O_3_, 730 nm for Si, and 865 nm for MoS_2_. For hBN, which shows no absorption in its reported range, we used the boundary of the available data (250 nm) [6]. We simulated square cross‐section waveguides on a SiO_2_ substrate (Figure 5a).

**FIGURE 5:**
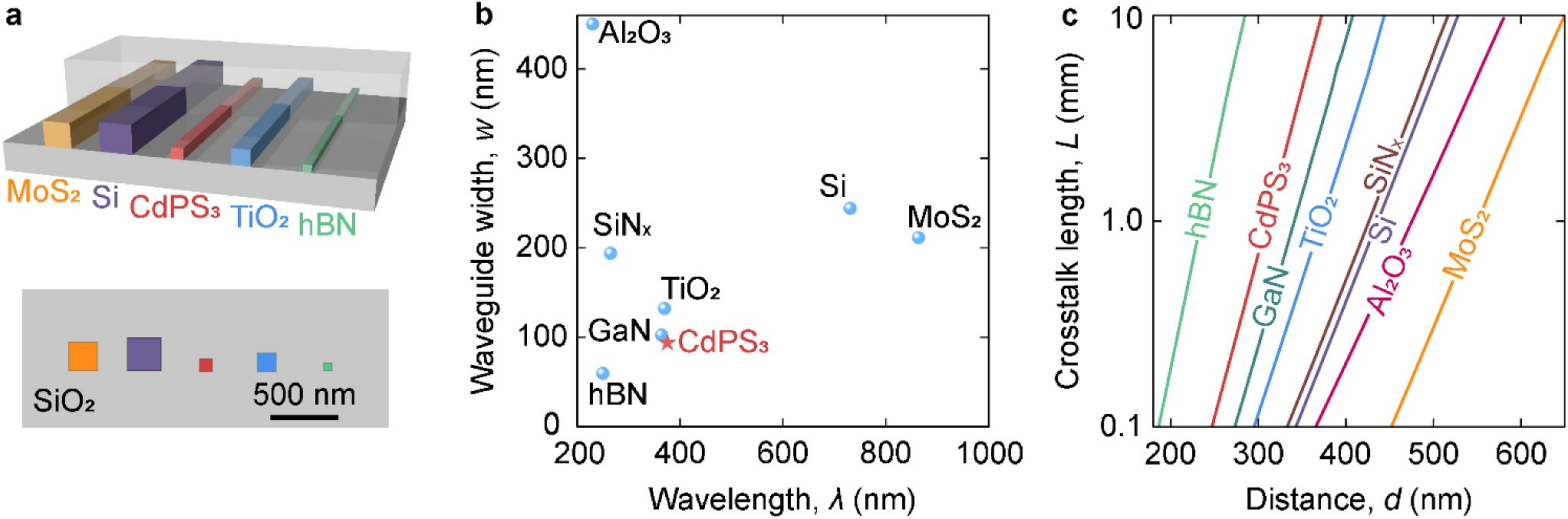
Prospect applications of CdPS_3_. (a) Schematic comparison of the dimensions of waveguides made from high‐refractive index materials: 59 × 59 nm^2^ for hBN, 132 × 132 nm^2^ for TiO_2_, 94 × 94 nm^2^ for CdPS_3_, 244 × 244 nm^2^ for Si, 211 × 211 nm^2^ for MoS_2_, 102 × 102 nm^2^ for GaN, 194 × 194 nm^2^ for SiN_x_ and 450 × 450 nm^2^ for Al_2_O_3_. (b) Waveguide width for different materials with cladding constant *χ* = 2.49 μm^−1^. (c) Relation of crosstalk length and distance between cores of the waveguides for high‐refractive index materials. The width is optimized for each given distance between waveguides.

To establish a fair comparison of confinement, we designed waveguides for each material such that the exponential decay constant (*χ*) of the evanescent field into the SiO_2_ cladding was fixed at *χ* = 2.49 μm^−1^. The decay constant relates to the effective mode index (*n*_eff_) and the cladding index (*n*_sub_) (see Methods). Figure 5b plots the required waveguide width (*w*) versus the operating wavelength (*λ*_min_). Because of its high refractive index, CdPS_3_ waveguide provides substantially tighter confinement (*w* = 94 nm at 375 nm) than TiO_2_ (*w* = 132 nm at 370 nm). On the other hand, silicon and MoS_2_, although offering high refractive indices, operate at much longer wavelengths, thus resulting in larger physical dimensions for the same relative confinement.

The ultimate limit of integration density is determined not only by mode confinement but also by evanescent field coupling (crosstalk) between adjacent waveguides. We calculated the distance required for power transfer between two adjacent waveguides (the crosstalk length, *L*), as a function of the separation distance *d* (Figure 5c) [26]. The waveguide widths were optimized to minimize crosstalk at each separation distance. These performance projections are based on fundamental intrinsic optical constants and assume perfectly smooth waveguide geometries. Although our broadband ellipsometry confirms a wide transparency window with the extinction coefficient *k* dropping below the instrumental sensitivity limit of ∼10^−3^, we are sure that in the significant part of this window optical losses fall much below 10^−3^, as the bulk crystal from which we exfoliated flakes is apparently transparent. This indicates that losses imposed by color centers are not prohibitive of chip‐scale waveguiding. In tightly confined systems, extrinsic factors such as Rayleigh scattering from surface and sidewall roughness induced during top‐down dry etching play a significant role. However, the competitive advantage of van der Waals materials is that they are atomically smooth, and etching techniques for these materials can produce atomically smooth sidewalls [38]. For this reason, we focused primarily on optical losses associated with the material's optical absorption. The results demonstrate that CdPS_3_ allows for significantly higher integration densities (shorter *d* for a given *L*) compared to TiO_2_ despite CdPS_3_'s higher operating wavelength. This analysis highlights the capability of CdPS_3_ to enable sub‐100 nm waveguides with high integration density at wavelengths inaccessible to conventional high‐index materials.

## 3 Conclusion

The search for high‐performing photonic materials often feels like a zero‐sum game. One could have either a wide transparency window that sacrifices refractive index, or a giant index locked behind the narrow gate of absorption. In this study, we show that this trade‐off is not a fundamental roadblock but a material challenge that can be solved. By looking at the MPX_3_ family we find in CdPS_3_ a surprisingly elegant solution. Our work provides a comprehensive study on its broadband optical properties, revealing a material that marries a near‐ideal high refractive index (*n* ≈ 3) with the wide bandgap for lossless operation in the blue part of the visible wavelength range. The immediate consequence is a new roadmap for device miniaturization. The dielectric tensor we report is not merely a catalog of properties; it is the essential blueprint for a new device architecture platform. It allows for a deterministic co‐design of material and light, enabling sub‐100 nm waveguides and meta‐atoms with performance metrics previously thought to be impossible for a transparent material.

## 4 Methods

### 4.1 Sample Preparation

Bulk CdPS_3_ single crystals were obtained from 2D Semiconductors Inc. and mechanically exfoliated onto Si, Si/SiO_2_, and Schott glass substrates at ambient conditions using adhesive scotch tapes (Nitto Denko Corporation). Before exfoliation, the substrates were sequentially treated with acetone, isopropanol, and deionized water, followed by exposure to air plasma to eliminate surface contaminants. For transmission electron microscopy (TEM) characterization, CdPS_3_ layers were transferred onto porous TEM grids (Norcada) via a dry‐transfer process assisted by a polydimethylsiloxane (PDMS) support layer. The thickness and surface morphology of CdPS_3_ flakes were characterized by an atomic force microscope (NT‐MDT Ntegra II) in HybriD mode at ambient conditions. Following the AFM characterization, only flakes with thicknesses exceeding 50 nm were selected for the subsequent measurements of optical properties to ensure a stable bulk‐like response.

### 4.2 Angle‐Resolved Raman Spectroscopy

Raman spectra were acquired with a Horiba LabRAM HR Evolution (HORIBA Ltd.) confocal microscope with × 100/N.A. = 0.90 objective, parallel analyzer and 1800 lines/mm diffraction grating were used. The studies were carried out at 632.8 nm excitation wavelength. For every polarization angle, Raman mapping was used to obtain an array of 9 spectra measured with 5 μm lateral step. Excitation power density was 125 kW/cm^2^, integration time was 10 s for each point, and spot area was 1.8 μm^2^. The spectra for each polarization angle were obtained by averaging all spectra of each map. The processing of the spectra was carried out by background subtraction. Afterward, background‐subtracted spectra were fitted by Lorentzian lines. Finally, results were used to fit the data and construct the polar graphs.

### 4.3 Angle‐Resolved Micro‐Spectroscopy

Polarization‐resolved transmittance measurements in the visible range were performed using a Zeiss Axio Lab.A1 optical microscope that was illuminated by a halogen light source. The instrument was coupled to an Optosky ATP5020P grating spectrometer using a Thorlabs M92L02 optical fiber with a 200 μm core diameter. Collection of the transmitted light was confined to a spot smaller than 15 μm by an “N‐Achroplan” 50 × Pol M27 objective lens, itself defined by a numerical aperture of 0.8. For reflection spectra, the same technical solution was employed, but with the use of an RX50M microscope and a SOPTOP MPlanFL, 50 ×, N.A. = 0.8 objective. The complete experimental configuration was detailed previously [39, 40]. Spectroscopic measurements were extended into the near‐ and mid‐infrared (NIR/MIR) regions using a Fourier transform infrared spectrometer (Bruker Vertex 80v) that was coupled to an infrared microscope (Hyperion 2000). Both reflectance and transmittance spectra were acquired at a normal incidence geometry using a 15 × reflective objective (N.A. = 0.4). The NIR band (900–1400 nm) was investigated using a configuration that included a tungsten–halogen lamp source, a calcium fluoride (CaF_2_) beamsplitter, and a nitrogen‐cooled mercury–cadmium–telluride (MCT) detector. To probe the MIR range (1400–17000 nm), a Globar thermal source and a potassium bromide (KBr) beamsplitter were employed. The optical spectra were modeled by solving the Fresnel equations within a two‐layer model, using tabulated substrate dispersion. For transmittance measurements on CaF_2_ and Schott glass, the thick substrates were modeled as incoherent layers. For reflectance measurements on single‐side polished Si substrates, backside reflections were neglected as the transmitted light was diffusely scattered by the rough back interface.

### 4.4 Spectroscopic Ellipsometry

Spectroscopic ellipsometry measurements of CdPS_3_ were performed using spectroscopic imaging ellipsometer Accurion EP4 in the rotating analyzer mode. Ellipsometry spectra were recorded for two flakes of CdPS_3_ with thicknesses of 50 and 165 nm. The spectral range of measurements is 250–1700 nm in 1 nm step and the incident angles of incidence are 45°, 50°, 55°, and 60°.

### 4.5 Scattering‐Type Scanning Near‐Field Optical Microscopy

For near‐field measurements we used two different scattering type scanning near‐field optical microscopes as well as several different source lasers. Measurements of thick flakes (∼1200 nm) were performed in mid‐infrared wavelengths using a NTEGRA nanoIR (NT‐MDT) scanning near‐field optical microscope. To excite the modes in that region we used QD5250C2, QD7500M1, and QD8500CM1 (Thorlabs) quantum cascade lasers working on 5219 nm, 7824 nm, and 8912 wavelengths, respectively. We used Pt‐coated silicon tip oscillating at the resonance frequency of *Ω* ≈ 185 kHz with an amplitude of 155 nm (NSG10/Pt). For the measurements of a flake (∼254 nm) in the visible—near‐infrared wavelengths region we used a NeaSNOM (Neaspec) s‐SNOM. As sources for that wavelength region we used Ti:Sapphire laser system (TiC, AVESTA) tunable from 700 to 1000 nm, operated at discrete wavelengths of 700 nm, 850 nm, and 1000 nm and a continuous wave Agilent 81600B tunable laser with a tunability range of 1475–1600 nm, operated at six discrete wavelengths with 25 nm step. We also employed various Pt/Ir‐coated silicon tips (ARROW‐NCPt‐50) oscillating at a resonance frequency of approximately *Ω* ≈ 280 kHz with an amplitude of 70–145 nm. For both wavelength ranges, the microscopes operated in reflection mode, using the same parabolic mirror for both excitation and collection of the near‐field signals. To improve the clarity of the near‐field images, we reduced the optical background by demodulating the detected signal at a higher‐order harmonic frequency *nΩ* (with *n* = 3 in our case), using an interferometric pseudoheterodyne detection scheme with a reference beam modulated by an oscillating mirror.

### 4.6 Numerical Calculations for Waveguides

Numerical calculations for waveguides with fixed decay constant and waveguides crosstalk length were performed using a coupled COMSOL Multiphysics and MATLAB environment via LiveLink. COMSOL was used as a solver, and for every task a simple 2D geometry model was used. MATLAB was used to control simulation parameters and manage the optimization process. To find the exact width of the waveguide for a given *χ*, we used a binary search algorithm. For optimizing widths of the waveguides for crosstalk calculations we employed a grid‐based parameter sweep followed by binary search refinement near the maximum.

### 4.7 First‐Principle Calculations

All ab initio calculations on optical properties of CdPS_3_ were done using VASP [41, 42]. Lattice parameters and atomic positions were taken from literature [27]. Theoretical results on the frequency‐dependent optical constants and electronic band structure were obtained within the single‐shot GW calculation based on the initial DFT run. Static dielectric constants were calculated within the standard DFT calculation. The kinetic energy with the plane waves basis set (ENCUT) was 400 eV in both steps. The convergence parameter of the self‐consistent electronic cycle (EDIFF) was 10^−8^ eV. Behavior of the core electrons was described with the projector augmented wave pseudopotentials (POTCAR) of GW type [43]. The exchange–correlation effects were described within the generalized gradient approximation (Perdew–Burke–Ernzerhof functional) [44]. The partial occupancies were set with the Gaussian method (ISMEAR = 0) with the smearing (SIGMA) of 0.05 eV. The first Brillouin zone (KPOINTS) was sampled with the 7 × 7 × 6 Γ‐centered grid. The total number of bands (NBANDS) considered in the both runs was 256. The number of points on the frequency grid in GW calculation (NOMEGA) was 200.

### 4.8 Transmission Electron Microscopy

The samples of CdPS_3_ were studied on a Titan Themis *Z* transmission (scanning) electron microscope, which allows studying the fine structure of the samples. The Titan Themis *Z* microscope is equipped with a sample corrector for correcting spherical aberrations, which significantly improves the resolution of the microscope.

### 4.9 Energy Dispersive X‐Ray Spectroscopy Analysis

The stoichiometry of the CdPS_3_ flakes was verified by energy dispersive X‐ray spectroscopy (EDX), which was performed using a Bruker QUANTAX EDX spectrometer integrated into a scanning electron microscope (SEM, JEOL JSM‐7001F) operating in the secondary electron imaging mode.

## Author Contributions

**Maksim R. Povolotskiy:** conceptualization, data curation, formal analysis, investigation, validation, visualization, writing – original draft, writing – review and editing. **Aleksandr S. Slavich:** conceptualization, data curation, formal analysis, investigation, validation, visualization, writing – original draft, writing – review and editing. **Georgy A. Ermolaev:** conceptualization, data curation, formal analysis, supervision, validation, visualization, writing – original draft, writing – review and editing. **Dmitriy V. Grudinin:** data curation, formal analysis, investigation, validation, visualization, writing – original draft, writing – review and editing. **Nikolay V. Pak:** investigation, writing – review and editing, visualization, formal analysis, data curation, validation. **Ilya A. Zavidovskiy:** data curation, formal analysis, investigation, validation, writing – review and editing, visualization. **Konstantin V. Kravtsov:** data curation, formal analysis, investigation, visualization, writing – review and editing, validation. **Anton A. Minnekhanov:** data curation, investigation, writing – review and editing, validation, formal analysis. **Mikhail K. Tatmyshevskiy:** data curation, formal analysis, investigation, validation, writing – review and editing. **Alexander V. Syuy:** data curation, investigation, validation, writing – review and editing. **Dmitry I. Yakubovsky:** data curation, formal analysis, investigation, validation, writing – review and editing. **Arslan Mazitov:** writing – review and editing, data curation, investigation, visualization. **Liudmila A. Klimova:** formal analysis, writing – review and editing. **Adilet N. Toksumakov:** writing – review and editing, investigation. **Alexander V. Melentev:** investigation, writing – review and editing, data curation. **Elena Zhukova:** investigation, supervision, writing – review and editing. **Davit A. Ghazaryan:** investigation, supervision, writing – review and editing. **Gleb Tselikov:** investigation, writing – review and editing, supervision. **Ivan Kruglov:** formal analysis, supervision, writing – review and editing. **Sergey M. Novikov:** investigation, writing – review and editing. **Andrey A. Vyshnevyy:** conceptualization, formal analysis, investigation, writing – review and editing. **Aleksey V. Arsenin:** conceptualization, funding acquisition, project administration, supervision, writing – review and editing. Kostya **S. Novoselov:** conceptualization, supervision, writing – review and editing, funding acquisition, project administration. **Valentyn S. Volkov:** conceptualization, funding acquisition, project administration, supervision, writing – review and editing.

## Conflicts of Interest

The authors declare no conflicts of interest.

## Supporting information


Supporting Information S1

## Data Availability

The data that support the findings of this study are available from the corresponding author upon reasonable request.
