# Supplementary material for: Record Index–Bandgap Trade‐Off: CdPS3 as a High‐Index van der Waals Platform for Ultraviolet–Visible Nanophotonics
Source: Nanophotonics. 2026 Jun 3;15(11):e70142. doi: 10.1002/nap2.70142 (PMC13642166; doi:10.1002/nap2.70142)
Supplement: Supplementary file 1 [file NAP2-15-e70142-s001.pdf]

Supplementary Information for

Record Index–Bandgap Trade-off: $CdPS_3$ as a High-Index van der Waals Platform for Ultraviolet–Visible Nanophotonics

Maksim R. Povolotskiy[1†], Aleksandr S. Slavich[2†], Georgy A. Ermolaev[2†], Dmitriy V. Grudinin[2], Nikolay V. Pak[1], Ilya A. Zavidovskiy[1], Konstantin V. Kravtsov[1,2], Anton A. Minnekhanov[2], Mikhail K. Tatmyshevskiy[1], Alexander V. Syuy[2], Dmitry I. Yakubovsky[1], Arslan Mazitov[2], Liudmila A. Klimova[2], Adilet N. Toksumakov[2], Alexander V. Melentev[1], Elena Zhukova[1], Davit A. Ghazaryan[3], Gleb Tselikov[2], Ivan Kruglov[2], Sergey M. Novikov[1], Andrey A. Vyshnevyy[2], Aleksey V. Arsenin[2], Kostya S. Novoselov[4,5,6], Valentyn S. Volkov[2*]

[1]Moscow Center for Advanced Studies, Kulakova str. 20, Moscow, 123592, Russia

[2]Emerging Technologies Research Center, XPANCEO, Internet City, Emmay Tower, Dubai, United Arab Emirates

[3]Laboratory of Advanced Functional Materials, Yerevan State University, Yerevan, 0025, Armenia

[4]National Graphene Institute (NGI), University of Manchester, Manchester, M13 9PL, UK

[5]Department of Materials Science and Engineering, National University of Singapore, Singapore, 03-09 EA, Singapore

[6]Institute for Functional Intelligent Materials, National University of Singapore, Singapore, 117544, Singapore

[†]These authors contributed equally to this work

*Correspondence should be addressed to e-mail: vsv@xpanceo.com

Table of Contents

Supplementary Note 1: Angle-resolved Raman spectroscopy

Supplementary Note 2: Spectroscopic ellipsometry of $CdPS_3$

Supplementary Note 3: First-principles calculations

Supplementary Note 4: Near-field optical microscopy data processing

Supplementary Note 5: Numerical procedure for waveguide width and crosstalk comparisons

Supplementary Note 1: Angle-resolved Raman spectroscopy

In the current study, analysis of the materials' vibrational response was carried out by polarized Raman measurements. For $CdPS_3$ with C2/m (C 1 2/m 1) space group, representation of Γ-point phonons is the following[1]:

$$\Gamma = \Gamma_{\text{acoustic}} + \Gamma_{\text{optic}} ,$$
$$\Gamma_{\text{acoustic}} = A_u + 2B_u ,$$
$$\Gamma_{\text{optic}} = 8A_g + 7B_g + 5A_u + 7B_u .$$

Among these phonons, A_g and B_g are Raman-active. In the current experimental setup, the linearly polarized beam is parallel to the interlayer direction.

For angle-resolved Raman spectroscopy, the A_g/B_g mode type determines the type of the polar plot. More specifically, the intensity of the vibrational mode is proportional to $\left|\boldsymbol{e}_s \hat{R} \boldsymbol{e}_L\right|^2$, where $\hat{R}$ is a Raman tensor of this mode, $\boldsymbol{e}_s$ and $\boldsymbol{e}_L$ are polarization vectors of scattered light and laser beam respectively. For A_g and B_g modes of monoclinic lattices with *b* being a unique axis, Raman tensors are represented by the following expressions:

$$\hat{R}(A_g) = \begin{pmatrix} a & 0 & d \\ 0 & b & 0 \\ d & 0 & c \end{pmatrix}, \hat{R}(B_g) = \begin{pmatrix} 0 & e & 0 \\ e & 0 & f \\ 0 & f & 0 \end{pmatrix},$$

For parallel configuration used in current study, this leads to the following expressions for angular dependencies of the intensities for Raman-active modes measured in parallel configuration:

$$I_{A_g}^{||}(\varphi) \sim (a\cos^2\varphi + b\ \sin^2\varphi)^2,$$

$$I_{B_g}^{||}(\varphi) \sim e^2 \sin^2 2\varphi ,$$

where φ is the angle between the polarization of the incident light and *a*-axis (i.e. $\boldsymbol{e}_L = \boldsymbol{e}_s = (\cos\varphi, \sin\varphi\ ,0)$.

The derived polar plots exhibit distinct dumbbell-like (for 223 cm^{-1}) and circular (for 247 and 377 cm^{-1}) patterns, confirming their assignment to A_g rather than B_g modes (the latter typically displaying "four-leaf clover" patterns).

To our knowledge, this work presents the first polarized Raman study of monoclinic $CdPS_3$. While the optical response of $CdPS_3$ is effectively isotropic within the basal plane, the vibrational spectrum reveals one prominent mode displaying a pronounced anisotropy. This distinct angular signature provides a reliable and non-destructive method for determining the in-plane crystallographic orientation of exfoliated flakes[2]. Moreover, $CdPS_3$ is theoretically predicted to possess anisotropic in-plane elastic stiffnesses and sound velocities[3], though these properties remain unexploited in functional devices. The ability to identify crystallographic axes via polarized Raman spectroscopy thus opens a practical pathway for harnessing this mechanical anisotropy, enabling the future realization of orientation-dependent phononic components such as directional acoustic waveguides, mechanical filters, and multi-frequency resonators.

To investigate the potential influence of local laser heating on the Raman spectra of the $CdPS_3$ flake, a power-dependent measurement series was conducted. Raman spectra were acquired from the same $CdPS_3$ flake over a wide range of excitation powers (0.05 mW to 4.5 mW), spanning powers both below and above the 2.2 mW used in the main text. Acquisition parameters were kept identical to those in the main text experiments: a 632.8 nm excitation wavelength, a ×100/N.A.=0.90 objective, and 1800 lines/mm diffraction grating.

The results of this power series are presented in Supplementary Figure 1. The spectra, normalized for clarity, show no measurable shift in the peak positions of any of the characteristic Raman modes across the entire power range. Furthermore, the line shapes and the relative intensities of the modes remain constant. Consequently, the Raman data and the conclusions drawn in the main text are confirmed to be free from laser-induced thermal variability.

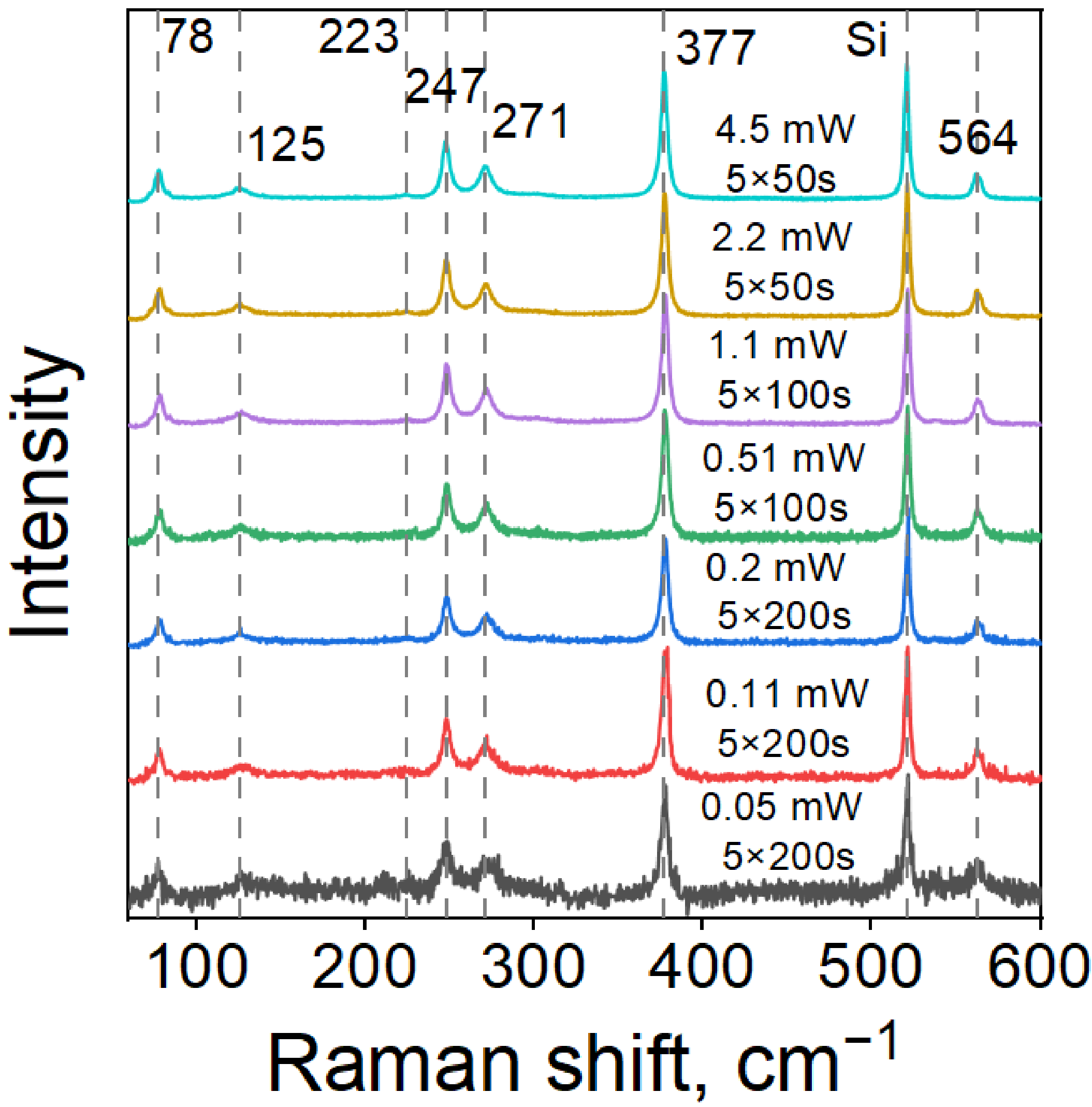

Supplementary Figure 1. Normalized power-independent Raman spectra demonstrating line positions and lineshape stability. Line positions indicated by numbers.

To confirm environmental stability of $CdPS_3$ under ambient conditions, we performed a time-dependent polarized Raman study on a selected flake over a period of 76 days. All measurements were conducted under standard laboratory atmosphere (room temperature, ambient humidity) without protective capping.

Spectra were acquired repeatedly over the 76-day period using a fixed excitation polarization geometry. The acquisition parameters were maintained constant across all measurements: 632.8 nm excitation wavelength, 4.5 mW excitation power, a 100× objective (N.A. = 0.90), a 1800 lines/mm diffraction grating, and a parallel analyzer configuration. Each spectrum represents an accumulation of 2 acquisitions of 50 seconds.

The resulting spectra are presented in Supplementary Figure 2. The data demonstrate that the key Raman line positions and spectral lineshapes remain essentially unchanged from week to week over the entire 10+ week period. No emergence of new peaks indicative of oxidation or hydration byproducts was observed. This negligible variability confirms the structural integrity and operational stability of $CdPS_3$ in air for the duration required for photonic characterization and integration procedures.

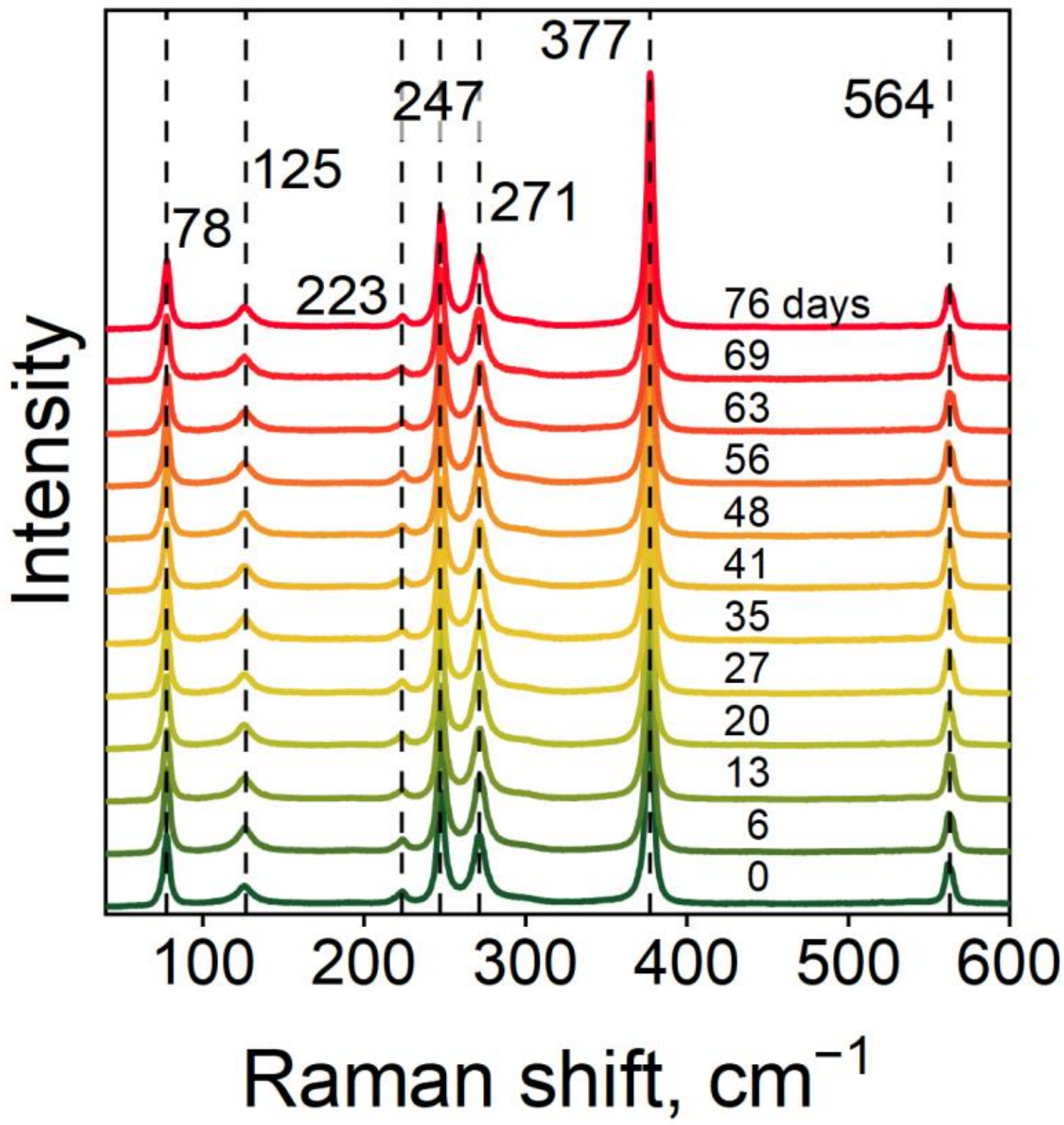

Supplementary Figure 2. Normalized Raman spectra obtained under a similar excitation polarization demonstrating line positions and lineshape stability over time. Line positions are indicated by numbers.

Supplementary Note 2: Spectroscopic ellipsometry of $CdPS_3$

We characterize the far-field anisotropic optical response of $CdPS_3$ through imaging ellipsometer Accurion EP4. To enhance the quality of our measurements we recorded the ellipsometry for two $CdPS_3$ flakes with different thicknesses of 50 nm (Supplementary Figure 3) and 165 nm (Supplementary Figure 4) on a standard SiO_2/Si substrate. For the description of in-plane optical properties, we use a traditional optical model for van der Waals materials, the Tauc–Lorentz oscillators[4], whose imaginary part of the dielectric permittivity for an individual oscillator is given by:

$$\varepsilon_2 = \begin{cases} \frac{1}{E} \cdot \frac{A \cdot E_0 \cdot C \cdot \left(E - E_g\right)^2}{\left(E^2 - E_0^2\right)^2 + C^2 E^2}, \text{for } E > E_g, \\ 0, \quad \text{otherwise} \end{cases} \tag{1}$$

where E is the photon energy, A is the strength of the oscillator, C is the broadening term, E_g is the optical bandgap, and E_0 is the peak central energy with the real part ε_1 of dielectric permittivity derived from the expression of ε_2 using the Kramers-Kronig relation plus $\varepsilon_{1,\infty}$. For the out-of-plane component of $CdPS_3$ dielectric tensor, we implement Sellmeier formula for the refractive index:

$$n = \sqrt{\frac{A_S \cdot \lambda^2}{\lambda^2 - \lambda_S^2}}, \tag{2}$$

where A_S is the amplitude of absorption, and λ_S is the center wavelength of absorption. The resulting parameters of the in-plane and out-of-plane parts of $CdPS_3$ dielectric tensor are collected in Supplementary Table 1.

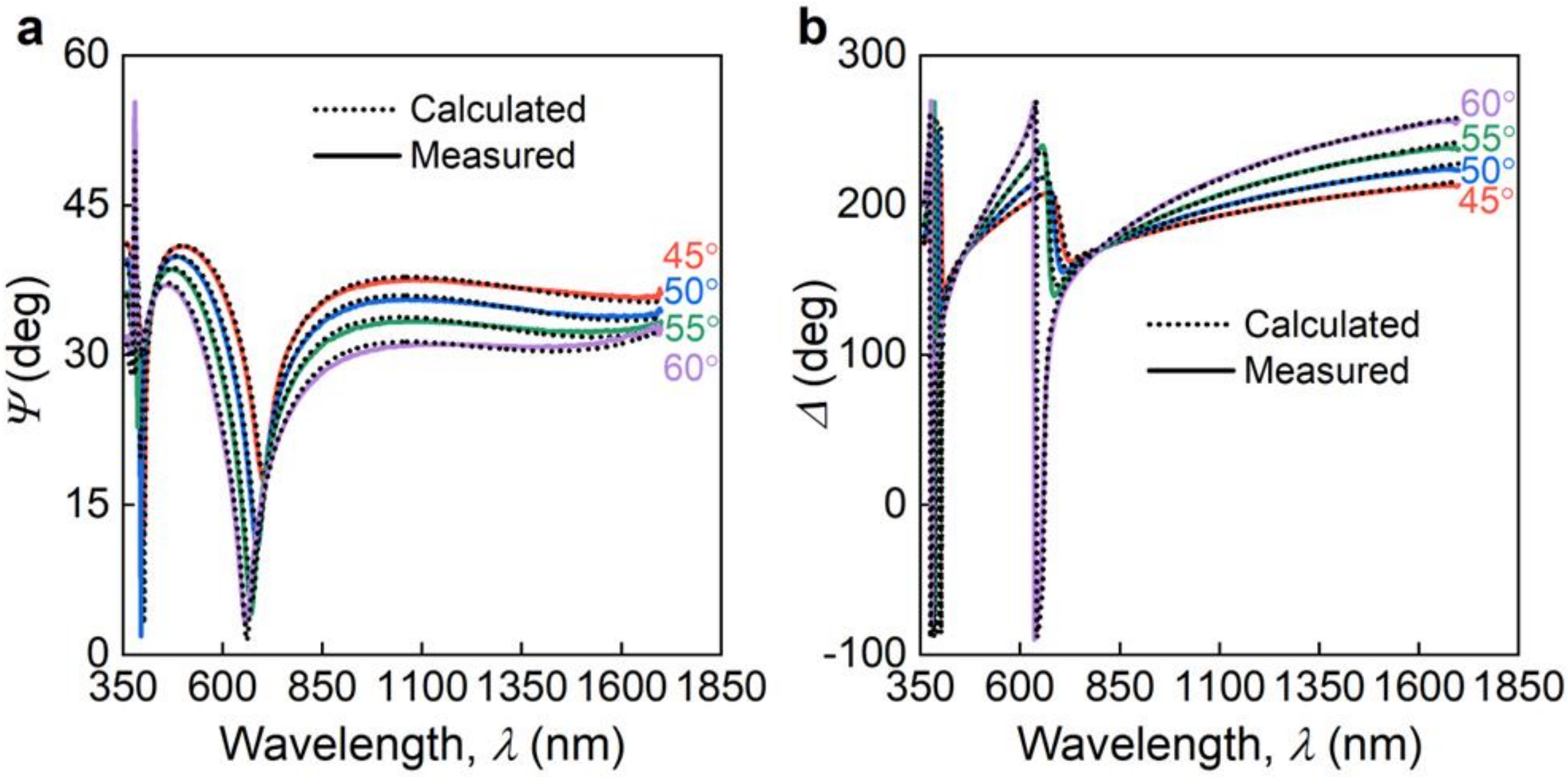

Supplementary Figure 3. Measured and calculated ellipsometric parameters **a,** Ψ and **b,** Δ for 50 nm-thick $CdPS_3$ on SiO_2(296 nm)/Si substrate.

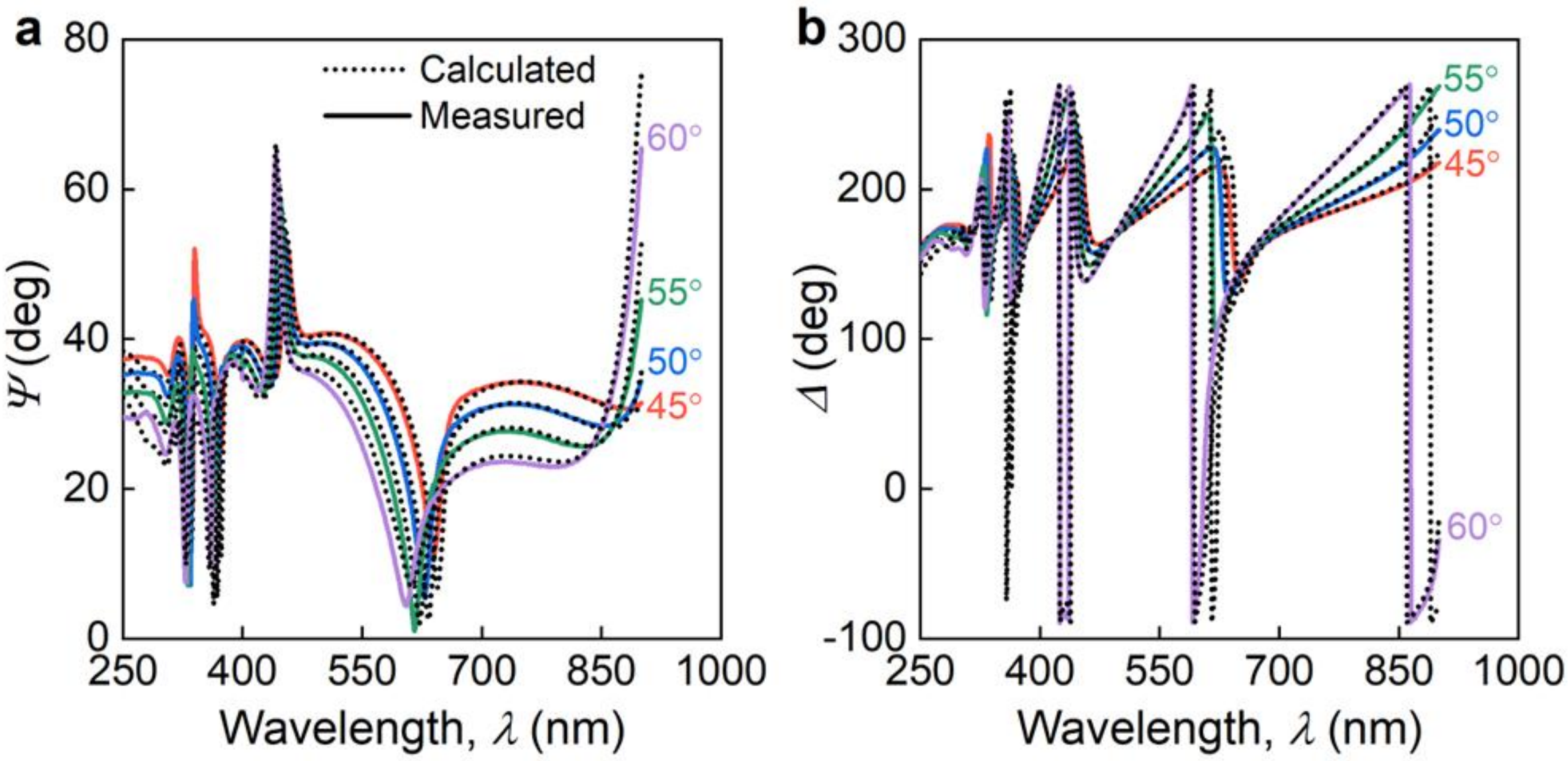

Supplementary Figure 4. Measured and calculated ellipsometric parameters **a,** Ψ and **b,** Δ for 165 nm-thick $CdPS_3$ on SiO_2(280 nm)/Si substrate.

Supplementary Table 1. Parameters of the in-plane and out-of-plane parts of $CdPS_3$ dielectric tensor: $\varepsilon_{1,\infty}$ = 3.840 ± 0.003, A_S = 4.345 ± 1.869, λ_S = 154.8 ± 5.4 nm.

Oscillator	A (eV)	E_0 (eV)	B (eV)	E_g (eV)
#1	148.05 ± 82.09	3.975 ± 0.038	0.100 ± 0.040	3.687 ± 0.648
#2	28.69 ± 1.03	4.253 ± 0.011	0.200 ± 0.007	3.670 ± 0.032
#3	84.27 ± 34.88	4.730 ± 0.007	0.785 ± 0.094	3.000 ± 0.050

To roughly estimate the upper limit of below bandgap optical absorption, we consider contributions of optically active defects (color centers). Color center absorption cross-section was taken as 10^{-17} cm^2 (see ref[5]), correspondingly. Without intentional doping in a high quality crystal, their concentration should not exceed 10^{18} cm^{-3} leading to the optical absorption of 10 cm^{-1} or below which corresponds to the extinction coefficient k~10^{-4}.

Supplementary Note 3: First-principles calculations

We computed the electronic band structure of $CdPS_3$ at the DFT (using PBE functional) and single-shot GW levels, with GW performed on top of the PBE ground state. The resulting GW band structures are shown in Supplementary Figure 5.

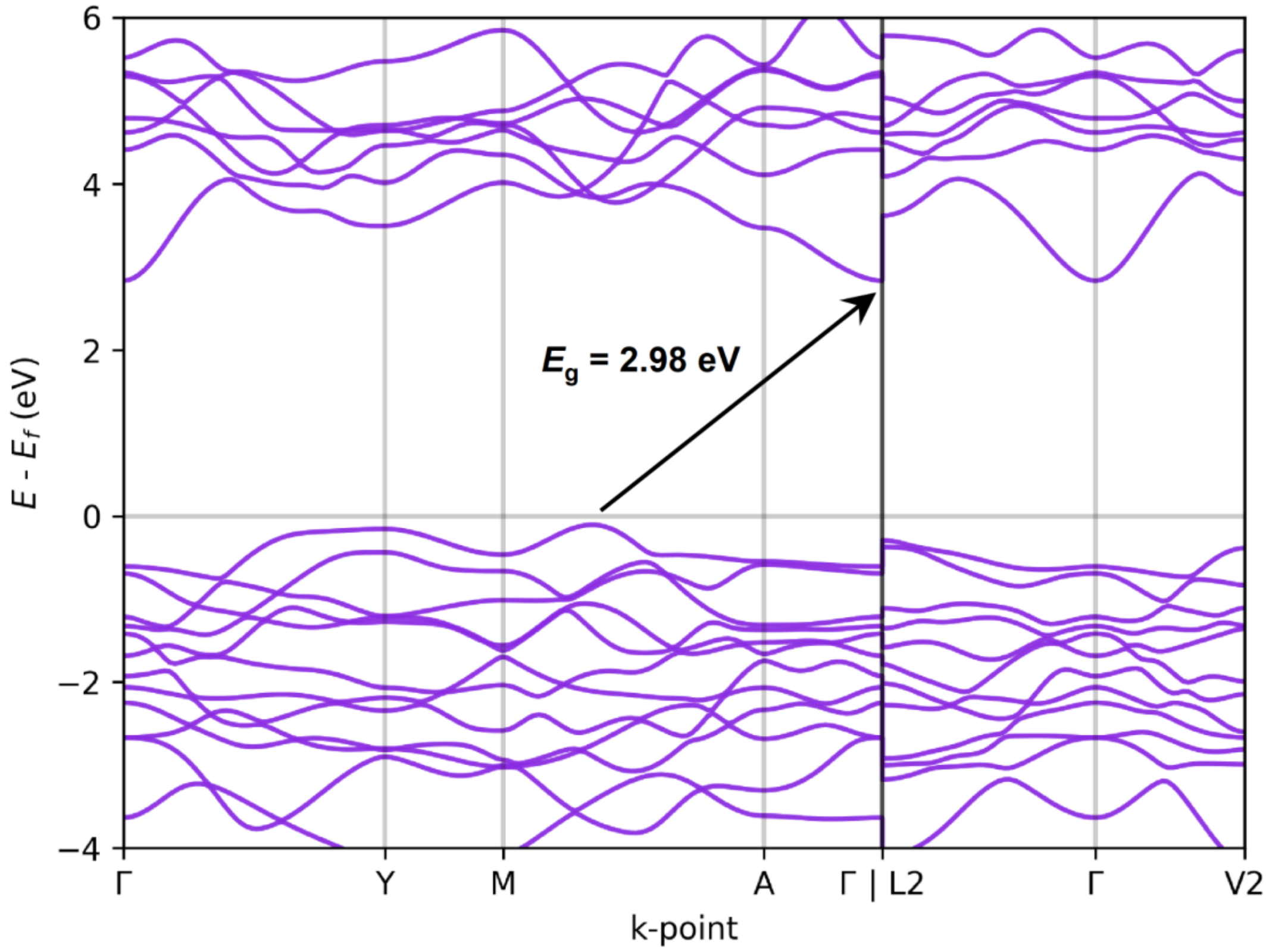

Supplementary Figure 5. Electronic band structure of $CdPS_3$ calculated within single-shot GW. Calculations confirm the indirect nature of the fundamental bandgap.

The PBE calculation significantly underestimates the bandgap, yielding a value of 1.80 eV. In contrast, the GW band structure confirms that $CdPS_3$ is an indirect-gap semiconductor. In particular, the GW calculation gives an indirect bandgap of 2.98 eV, with the valence-band maximum located along the M-A direction of the Brillouin zone and the conduction-band minimum at the Γ point. The lowest direct transition is found at the Γ point and its energy amounts to 3.54 eV. These values are in agreement with previous theoretical and experimental reports for $CdPS_3$.

The calculated band dispersions also help clarify the weak in-plane optical anisotropy discussed in the main text. As shown in Supplementary Figure 6, the GW dispersions along the two in-plane reciprocal directions, Γ-B1 and Γ-B2, are nearly identical, whereas the dispersion along the out-of-plane direction shows a different behavior. This result is consistent with the experimentally observed nearly isotropic in-plane optical response and indicates that the relevant electronic transitions contribute very similarly to the two in-plane components of the dielectric function. In this sense, the near-isotropic in-plane optical behavior is not accidental, but reflects a very weak anisotropy of the electronic band structure.

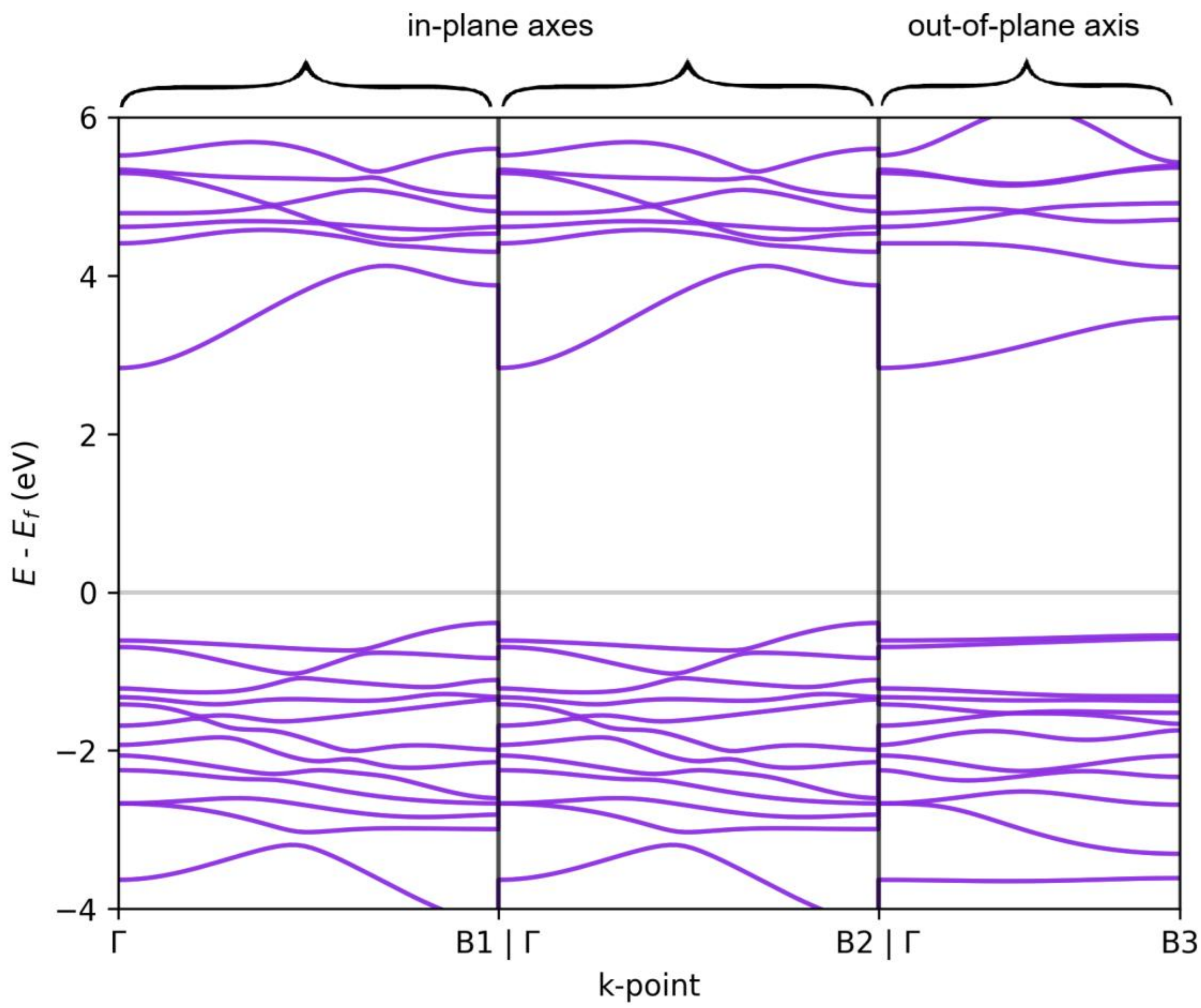

Supplementary Figure 6. GW band structure of $CdPS_3$ plotted along three reciprocal directions in the first Brillouin zone, B1, B2, and B3. The dispersions along Γ-B1 and Γ-B2 are nearly identical, indicating a very weak in-plane anisotropy of the electronic structure. This is consistent with the nearly isotropic in-plane optical response discussed in the main text.

At the same time, this should not be interpreted as exact symmetry-enforced in-plane isotropy. $CdPS_3$ remains a monoclinic crystal, and its Raman response shows both anisotropic and nearly polarization-independent vibrational modes. This is not contradictory, since Raman anisotropy is governed by vibrational Raman tensors, whereas the optical response discussed here is determined by electronic transitions.

The present single-shot GW approach does not explicitly include excitonic effects. Therefore, while the calculations correctly reproduce the overall dispersion trends and the indirect nature of the gap, exact quantitative agreement with the experimental optical constants is not expected, particularly near the absorption edge and in the UV spectral region.

Supplementary Note 4: Near-field optical microscopy data processing

Scanning near-field optical microscopy (SNOM) is based on detection of the scattered evanescent light waves in a close vicinity of a sample's surface with a probe, which allows to depict modes propagating in the crystal in different directions. Figure 4 of the main text illustrates such as-taken maps, with the alternating constructive and destructive interference regions being observed as a result of the interference of the background signal collected as a result of direct reflection of the light at the probe-sample interface and the traveling through the sample mode that was excited on the edge of the sample and scattered via the probe. This interference causes the Fourier transform to yield a shift in the observed effective mode index for the traveling modes. The shift value is related to the geometry of the experiment and can be described as[6,7]:

$$n_{\mathrm{eff}} = n_{\mathrm{SNOM}} \cos\beta + \cos\alpha \sin(\beta + \theta),$$

where α and ϑ are the incident angles with respect to horizontal plane and sample edge respectively, n_{eff} is the effective index of the mode propagating inside planar $CdPS_3$ waveguide, n_{SNOM} is the effective index obtained from FFT obtained from near-field measurements and β is the angle that the traveling direction of the waveguide mode makes with normal direction of sample edge, which satisfies the following momentum conservation equation:

$$\sin\beta = \frac{\cos\alpha \cos\theta}{n_{eff}}$$

In case of our measurements the orientation of the sample was chosen so θ=90°±5° and α = 25°±5°. For this geometry the relation between the effective index extracted from the Fourier transform of the near-field map (n_{SNOM}) and the actual modal effective index simplifies to:

$$n_{\mathrm{eff}} = n_{\mathrm{SNOM}} + \cos\alpha$$

The shift value extracted from the geometry of the experiment can be verified by the fact that the observable effective index of the air mode is shifted to the position near $n_{\mathrm{eff}} = 1$. The uncertainty in the extracted effective index arises from the uncertainty of the Fourier peak position and from the uncertainty in the geometric correction due to the angular alignment. The latter propagates according to:

$$\Delta n_{geom} = cos\,(\alpha)\delta\alpha$$

The total uncertainty of the reported effective indices is therefore estimated as:

$$\Delta n_{eff} = \Delta n_{SNOM} + cos\,(\alpha)\delta\alpha$$

Near-field amplitude and phase images of the 254 nm thick $CdPS_3$ flake acquired at wavelengths of 700, 850, and 1000 nm are shown in Supplementary Figure 7a, together with the corresponding line profiles (Supplementary Figure 7b) and their Fourier transforms (Supplementary Figure 7c). The Fourier spectra exhibit clear peaks corresponding to guided modes supported by the slab, enabling extraction of their effective mode indices.

Across the entire visible–NIR spectral range investigated, we consistently observe signatures of the fundamental transverse-electric (TE_0) and transverse-magnetic (TM_0) modes, as well as the light line (air mode). The experimentally extracted dispersion of these modes is in good agreement with transfer matrix method (TMM) calculations (Supplementary Figure 8), confirming their assignment.

In contrast, no additional peaks attributable to higher-order guided modes (TE_1 or TM_1) are observed in the Fourier spectra at any wavelength. This includes the 700 nm case, where TMM calculations predict the presence of a TE_1 mode within the accessible momentum range. Specifically, no discernible spectral features are detected near the expected in-plane momentum of the TE_1 branch, indicating that this mode does not contribute measurably to the near-field signal.

The absence of observable higher-order modes can be understood from their modal properties. TMM and eigenmode analysis show that the TE_1 mode possesses a lower effective index compared to the fundamental modes, placing it closer to the light line. As a result, this mode is more weakly confined within the flake, with a substantial fraction of its field extending into the surrounding media. Such reduced confinement leads to two key effects: (i) inefficient excitation by the near-field tip, which preferentially couples to strongly confined, high-momentum modes, and (ii) a diminished near-field amplitude at the sample surface, resulting in weak or absent interference fringes in real space. Consequently, the contribution of the TE_1 mode to the measured signal is strongly suppressed and falls below the detection threshold of the experiment.

Overall, while TMM predicts the onset of a TE_1 mode at shorter wavelengths (≈700 nm), our measurements demonstrate that only the fundamental TE_0 and TM_0 modes are experimentally accessible in the 254 nm flake. The effective indices of these modes agree well with theoretical predictions across the visible–NIR window, whereas no measurable signature of TE_1 or TM_1 is detected.

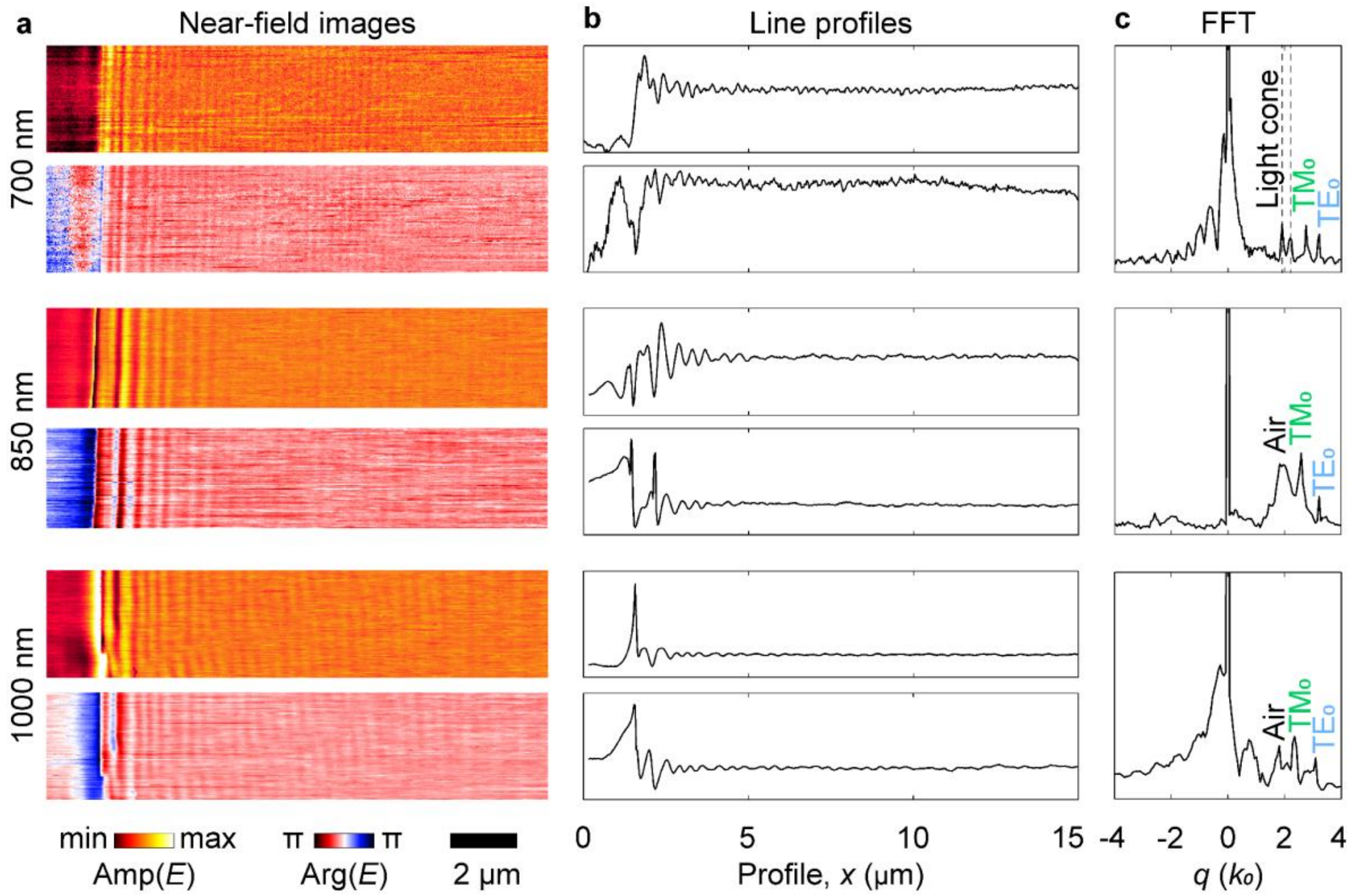

Supplementary Figure 7. Near-field measurements of $CdPS_3$ 254 nm flake. **a** Near-field images, amplitude Amp(E) and phase Arg(E), of the electric field E taken at 700 nm (top), 850 nm and 1000 nm (bottom). **b** x-Line scans taken from panel **a**. **c** Fast Fourier transform (FFT) amplitude of the complex near-field signal in **b**.

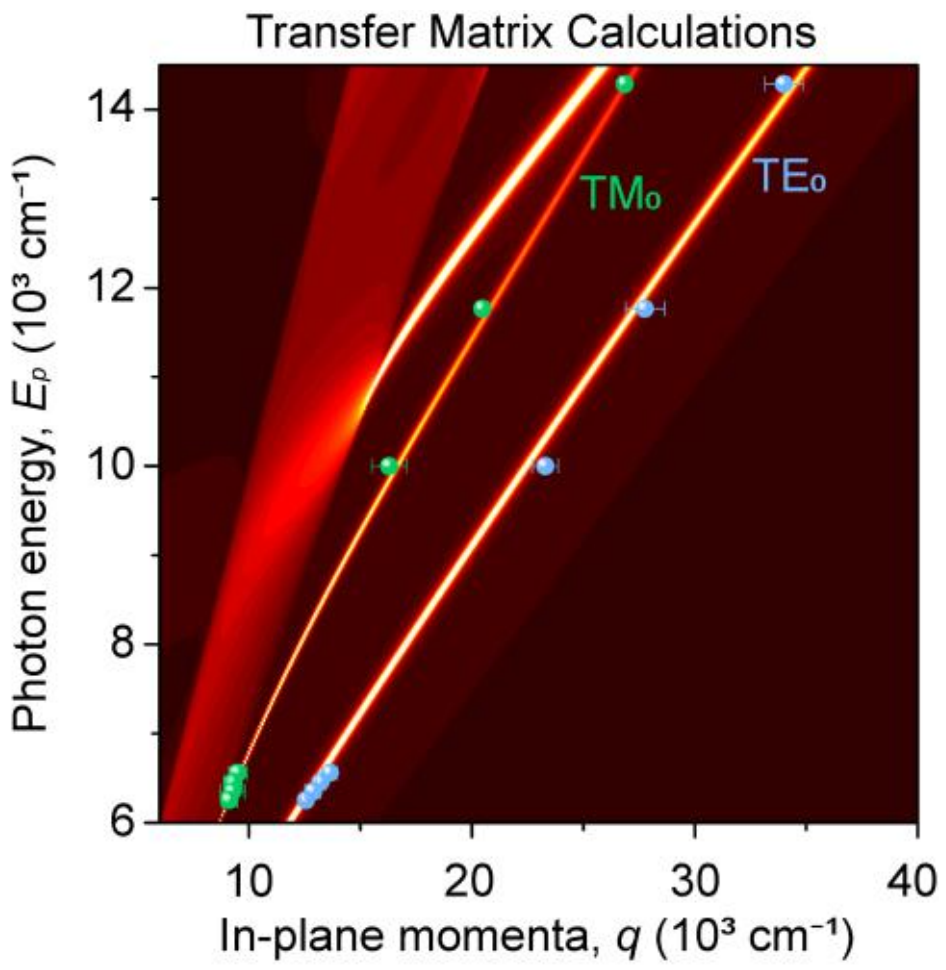

Supplementary Figure 8. Transfer Matrix Calculations for air/$CdPS_3$(254 nm)/CaF_2.

Supplementary Note 5: Numerical procedure for waveguide width and crosstalk comparisons

Numerical simulations were carried out in a coupled COMSOL Multiphysics–MATLAB environment, using COMSOL as the eigenmode solver and MATLAB for automated parameter control and optimization. All calculations assumed waveguides fully encapsulated in SiO_2, with the refractive index of the cladding denoted n sub. For each material, refractive indices and extinction coefficients at the minimal extinction wavelength λ_{min} (defined by k=0.01) were taken from reference [8] for hBN, TiO_2, Si, and MoS_2, and from the present work for $CdPS_3$. The dielectric tensor was taken as constant at λ_{min} for each material. Square cross-sections were considered throughout, with waveguide height equal to width.

For confinement analysis (Figure 5b), the effective width w was determined for waveguides exhibiting a fixed exponential decay constant χ=2.49 µm^{-1} of the mode field in the cladding. Here χ is defined as

$$\chi = \sqrt{{n_{\mathrm{eff}}}^2 - {n_{\mathrm{sub}}}^2} * 2\pi/\lambda_{\mathrm{min}}$$

where n_{eff} is the effective mode index of the guided mode. For each material at its λ_{min}, the waveguide width w satisfying the target χ was found using a binary search algorithm over COMSOL-calculated eigenmodes. This approach ensured a direct comparison of field confinement across materials at wavelengths where each remains below the global permissible loss threshold.

For integration density analysis (Figure 5c), the crosstalk distance L between two identical parallel waveguides separated laterally by distance d was evaluated using:

$$L = \frac{\lambda_{\mathrm{min}}}{2|n_o - n_e|}$$

where n_o and n_e are the effective indices of the odd and even supermodes, respectively, of the coupled system. At each separation d and for each material at its λ_{min}, the waveguide width was optimized to maximize L, using a coarse grid-based sweep followed by binary search refinement near the optimum. This procedure isolates material-dependent performance from purely geometric scaling effects.

The combined results demonstrate that $CdPS_3$ supports both tight confinement at fixed decay constant and large crosstalk distances despite its relatively long λ_{min}, owing to its high refractive index and strong optical anisotropy.

References

1. Kroumova, E. *et al.* Bilbao crystallographic server: Useful databases and tools for phase-transition studies. *Phase Transitions* **76**, 155–170 (2003).
2. Slavich, A. S. *et al.* Germanium disulfide as an alternative high refractive index and transparent material for UV-visible nanophotonics. *Light Sci. Appl.* **14**, 213 (2025).
3. Reed, B. W. & Koski, K. J. Acoustic phonons and elastic stiffnesses from Brillouin scattering of CdPS3. *J. Appl. Phys.* **131**, 165109 (2022).
4. Ermolaev, G.A. *et al.* Giant optical anisotropy in transition metal dichalcogenides for next-generation photonics. *Nat. Commun.* **12**, 854 (2021).
5. Fraczek, E. et al. Laser spectroscopy of NV- and NV0 colour centres in synthetic diamond. Opt. Mater. Express 7, 2571 (2017).
6. Hu, D. *et al.* Probing optical anisotropy of nanometer-thin van der waals microcrystals by near-field imaging. *Nat. Commun.* **8**, 1471 (2017).
7. Sternbach, A. J. *et al.* Femtosecond exciton dynamics in WSe2 optical waveguides. *Nat. Commun.* **11**, 3567 (2020).
8. Grudinin, D. V. *et al.* Hexagonal boron nitride nanophotonics: a record-breaking material for the ultraviolet and visible spectral ranges. *Mater Horiz* **10**, 2427–2435 (2023).
